# Protocols for Oral Infection of Lepidopteran Larvae with Baculovirus

**DOI:** 10.3791/888

**Published:** 2008-09-03

**Authors:** Wendy Sparks, Huarong Li, Bryony Bonning

**Affiliations:** Department of Entomology, Iowa State University

## Abstract

Baculoviruses are widely used both as protein expression vectors and as insect pest control agents. This video shows how lepidopteran larvae can be infected with polyhedra by droplet feeding and diet plug-based bioassays. This accompanying Springer Protocols section provides an overview of the baculovirus lifecycle and use of baculoviruses as insecticidal agents, including discussion of the pros and cons for use of baculoviruses as insecticides, and progress made in genetic enhancement of baculoviruses for improved insecticidal efficacy.

**Figure:**
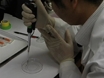


## Protocol

Please visit Springer Protocols to learn more about the engineering baculovirus as an insecidical agent and the oral infection techniques used in this assay.

